# Systemic Simvastatin Rescues Retinal Ganglion Cells from Optic Nerve Injury Possibly through Suppression of Astroglial NF-κB Activation

**DOI:** 10.1371/journal.pone.0084387

**Published:** 2014-01-02

**Authors:** Seita Morishita, Hidehiro Oku, Taeko Horie, Masahiro Tonari, Teruyo Kida, Akiko Okubo, Tetsuya Sugiyama, Shinji Takai, Hideaki Hara, Tsunehiko Ikeda

**Affiliations:** 1 Department of Ophthalmology, Osaka Medical College, Osaka, Japan; 2 Department of Pharmacology, Osaka Medical College, Osaka, Japan; 3 Molecular Pharmacology, Department of Biofunctional Evaluation, Gifu Pharmaceutical University, Gifu, Japan; Dalhousie University, Canada

## Abstract

Neuroinflammation is involved in the death of retinal ganglion cells (RGCs) after optic nerve injury. The purpose of this study was to determine whether systemic simvastatin can suppress neuroinflammation in the optic nerve and rescue RGCs after the optic nerve is crushed. Simvastatin or its vehicle was given through an osmotic minipump beginning one week prior to the crushing. Immunohistochemistry and real-time PCR were used to determine the degree of neuroinflammation on day 3 after the crushing. The density of RGCs was determined in Tuj-1 stained retinal flat mounts on day 7. The effect of simvastain on the TNF-α-induced NF-κB activation was determined in cultured optic nerve astrocytes. On day 3, CD68-positive cells, most likely microglia/macrophages, were accumulated at the crushed site. Phosphorylated NF-κB was detected in some astrocytes at the border of the lesion where the immunoreactivity to MCP-1 was intensified. There was an increase in the mRNA levels of the *CD68* (11.4-fold), *MCP-1* (22.6-fold), *ET-1* (2.3-fold), *GFAP* (1.6-fold), *TNF-α* (7.0-fold), and *iNOS* (14.8-fold) genes on day 3. Systemic simvastatin significantly reduced these changes. The mean ± SD number of RGCs was 1816.3±232.6/mm^2^ (n = 6) in the sham controls which was significantly reduced to 831.4±202.5/mm^2^ (n = 9) on day 7 after the optic nerve was crushed. This reduction was significantly suppressed to 1169.2±201.3/mm^2^ (*P* = 0.01, Scheffe; n = 9) after systemic simvastatin. Simvastatin (1.0 µM) significantly reduced the TNF-α-induced NF-κB activation in cultured optic nerve astrocytes. We conclude that systemic simvastatin can reduce the death of RGCs induced by crushing the optic nerve possibly by suppressing astroglial NF-κB activation.

## Introduction

Reactive astrogliosis is an important step in the repair process of cells in the central nervous system (CNS) after different types of injuries [1]. It is usually accompanied by neuroinflammation which can cause more neuronal damage [2]. For example, reactive astrocytes express several cell adhesion molecules [3] and chemokines [4] which facilitate the infiltration of leucocytes and macrophages to the injured site. In addition, reactive astrocytes in concert with the recruited microglia/macrophages induce the formation of pro-inflammatory molecules including TNF-α and nitric oxide [5].

Statins, inhibitors of HMG-CoA reductase, are widely used as cholesterol-lowering drugs. However, statins also have neuro-protective properties, and they have been shown to rescue retinal ganglion cells (RGCs) from excitotoxicity [6], retinal ischemia [7]–[10], and optic nerve injury [11]. Statins inhibit the induction of nitric oxide synthase (NOS) in glial cells [12] and suppress the accumulation of leukocytes in lesions [6].

Crushing the optic nerve is commonly used to study neurodegenerative processes in the optic nerve and retina. In earlier studies, we demonstrated that neuroinflammation in the optic nerve played a critical role in the death of RGCs after the optic nerve was crushed [13]. We found that microglia/macrophages that were immunopositive to CD68 were recruited to the crushed site of the optic nerve. These cells expressed endothelin-B (ETB) receptors, secreted endothelins (ETs), and activated astroglia. In addition, pro-inflammatory genes such as *TNF-α* and inducible nitric oxide synthase (*iNOS*) were up-regulated in the optic nerve. An inhibition of the ETB receptors by BQ-788 protected the RGCs from death after the optic nerve crush, and this protection was found to be due to the suppression of the pro-inflammatory genes.

Lipophilic simvastatin can cross the blood-brain barrier and systemic administration of simvastin has been shown to reduce the 1-methyl-4-phenyl-1,2,3,6-tetrahydropyiridine (MPTP)-induced neurotoxicity [14] and to rescue neurons from traumatic brain injury [15]. Statins have also been shown to suppress astroglial NF-κB activation [14] which is required for the transcription of different pro-inflammatory molecules [16]–[18] and chemokines including monocyte chemoattractant protein-1 (MCP-1) in astrocytes [19]. These findings suggested that the neuro-protective effect of statins was most likely due to their anti-inflammatory property. Although the neuroprotective effects of statins have been extensively examined in the retina [6]–[11], the effects of systemic statins on the induction of reactive astrogliosis and its related neuroinflammation in the optic nerve have not been determined.

Because statins were found to rescue RGCs from optic nerve injury, we hypothesized that this protective effect was due to the ability of simvastatin to suppress the activation of optic nerve astroglia. To test this hypothesis, we crushed the optic nerves of rats that were given either simvastatin or vehicle by an osmotic minipump. The effects of systemic simvastatin on the astroglial activation and related changes in the optic nerve were determined by immunohistochemistry. In addition, real-time PCR was performed to determine the changes of the *CD68*, *MCP-1*, *ET-1*, *GFAP*, *TNF-α*, and *iNOS* genes to monitor the degree of neuroinflammation in the optic nerve. The inhibitory effect of simvastatin on the astroglial activation of NF-κB was determined in cultured optic nerve astrocytes.

## Methods

### Animals

Nine-week old male Wistar rats were purchased from Japan SLC (Shizuoka, Japan). The rats were housed in an air-conditioned room with a temperature of approximately 23°C, humidity of 60%, and the room lights on a 12∶12 light:dark cycle. All animals were handled in accordance with the ARVO resolution for the Use of Animals in Ophthalmic and Vision Research. The experimental protocol was approved by the Committee of Animal Use and Care of Osaka Medical College (No. 25074). A total of 85 adult rats were used.

### Chemicals

Unless noted, chemicals were purchased from Sigma-Aldrich (St. Louis, MO, USA). An NF-κB activation inhibitor, 6-amino-4(4-phenoxyphenylethylamino) quinazoline (QNZ), was purchased from Merck Chemicals (Darmstadt, Germany).

### Optic Nerve Crush

Animals were anesthetized with an intraperitoneal injection of pentobarbital sodium. A skin incision was made along the midline of the skull to expose the superior surface of the right eye. The superior rectus muscle was incised to expose the optic nerve, and the optic nerve was crushed with forceps 2 mm behind the eye for 10 seconds [20]. Care was taken not to occlude the blood vessels and cause retinal ischemia. We confirmed that the retinal circulation was not blocked by indirect ophthalmoscopy [21] and have verified that the HIF-1α gene was not up-regulated by real-time PCR [13]. A sham operation was performed on the right eyes of other animals, and the optic nerve was exposed in the same way but not crushed as in the experimental animals. The left eyes were not used as controls because it has been demonstrated that crushing one optic nerve can affect the morphology of the contralateral retina [22].

In some animals, simvastatin (1.0 mg/kg/day, Tokyo Chemical, Tokyo, Japan) or its vehicle (polyethylene glycol, Wako, Osaka, Japan) was given via osmotic minipumps (DURECT, Cupertino, CA, USA), which were placed beneath the abdominal skin one week prior to crushing the optic nerves. This concentration was chosen because simvastatin at 1.0 mg/kg/day had neuroprotective effects on the brain from traumatic injury [23] and MPTP-induced neurotoxicity [14].

### Quantitative RT-PCR Analysis

We determined the changes in the expression of several genes in the optic nerves by real-time PCR (RT-PCR). The genes studied were the *CD68*, *MCP-1*, *ET-1*, *GFAP*, *TNF-α*, and *iNOS.* In addition, changes of the *RhoA* gene in the optic nerves were determined to monitor the bioavailability of simvastatin in the optic nerve. In preliminarily experiments, we found by RT-PCR that the peak expression of *CD68* in the optic nerve was on days 3 to 5. Thus, the animals were killed on day 3.

Approximately 4 mm of the optic nerve centered on the crush site was incised. The optic nerves were homogenized in lysis buffer, and the RNA was extracted with the RNeasy plus mini kit (QIAGEN, Valencia, CA, USA). The RNA concentrations and purity were calculated from the absorbance at 260/280 nm.

The RNA was reverse transcribed with PrimeScript reverse transcriptase reagent (Takara, Ohtsu, Shiga, Japan). The cDNA was used for quantitative real-time PCR amplification with the TaqMan Gene Expression Assays for the targeted genes (Applied Biosystems, Foster City, CA, USA). Rat TaqMan Gene Assays for CD68 Rn01495634_g1, MCP-1 (CCL2) Rn00580555_m1, ET1 Rn00561129_m1, GFAP Rn00566603_m1, TNF-α Rn01525859_g1, iNOS (NOS2) Rn00561646_m1, and RhoA Rn04219609_m1 were used. Amplicons were detected using the relevant probes tagged with MGB quencher and FAM dye. TaqMan rat 18s rRNA control Expression Assays (Applied Biosystems) were used as the reference genes.

Real-time PCR was performed in Premix Ex Taq (Perfect Real Time; Takara). All reactions were run on a Thermal Cycler Dice Real time system TP870 (Takara) with the following cycling parameters: 30 s at 95°C followed by 40 cycles at 95°C for 5 s, and 60°C for 30 s. A standard curve of the cycle thresholds was established using serial dilutions of cDNA samples. The targeted gene values were normalized to the relative amounts of 18s rRNA.

### Immunohistochemistry

On day 3, rats were deeply anesthetized with an intraperitoneal injection of pentobarbital sodium and perfused through the heart with saline followed by 4% paraformaldehyde (PFA) in 0.1 M phosphate buffer of pH 7.4. After removing the skull and cerebral hemispheres, the optic nerves and the eyes were carefully removed and post-fixed in 4% PFA in PBS overnight. These tissues were used for immunohistochemistry. After washing with PBS, the tissues were immersed in 30% sucrose overnight at 4°C and then embedded in OCT compound (BDH Laboratory Supplies, Poole, UK). Then, 10 µm frozen sections were cut with a cryostat. After blocking with 1.0% normal goat or donkey serum plus 1.0% BSA and 0.1% triton-X 100 in PBS, the sections of the optic nerves were incubated with primary antibodies of mouse anti-CD68 antibody (1∶500, Serotec, Oxford, UK), goat polyclonal anti-MCP-1 antibody (1∶200, Santa Cruz, Dallas, Texas, USA), rabbit polyclonal anti-GFAP (1∶500, Merck Millipore, Billerica, MA, USA), or mouse monoclonal anti-GFAP antibody (1∶500, Sigma) overnight at 4°C. In addition, some sections were incubated with rabbit polyclonal anti-phosphorylated NF-κB p65 (1∶200, Cell Signaling, Beverly, MA, USA) and rabbit polyclonal anti-TNF-α (1∶500, Bioss, Boston, MA, USA) to determine NF-κB activation and involvement of TNF-α in the activation. These sections were incubated for 2 hrs at room temperature in Alexa 594 or Alexa 488-conjugated to the appropriate secondary antibodies (Invitrogen, Carlsbad, CA, USA) diluted by 1∶500.

The processed sections were photographed with a fluorescent microscope (BZ 8000, Keyence, Osaka, Japan) or a confocal laser microscope (TCS SP8, Leica, Wetzlar, Germany).

### Labeling Retinal Ganglion Cells

A loss of RGCs is known to occur in a delayed fashion after crushing the optic nerve; the number of RGCs remains unchanged for 5 days and then abruptly decreases to 50% on day 7 and to less than 10% on day 14 [21]. Thus, the loss of RGCs was determined on day 7 after crushing the optic nerve.

To study the effects of crushing the optic nerves, rats were killed on day 7 and the retinas were carefully removed from the eyes as described in detail by Winkler [24]. In brief, rats were euthanized by CO_2_, and the globe was proptosed by placing forceps around the optic nerve just behind the eyeball. The globe was transected along the equator and the cornea and lens were removed. The retina was detached from the pigment epithelium by pressing upward with the forceps and removed by cutting its attachment to the optic nerve head. The isolated retina was placed in PBS solution immediately, and any vitreous remaining on the isolated retina was carefully removed.

The retinas were then flat mounted, sandwiched between nylon mesh sheets, and fixed in 4% PFA in PBS overnight at 4°C. After washing in PBS and blocking in PBS containing 1.0% BS and 0.3% triton X-100, the retinas were incubated with Alexa 488-conjugated mouse monoclonal neuron-specific class III beta-tubulin (Tuj-1) antibody (Covance, Princeton, NJ, USA) (1∶500). Tuj-1 is a specific marker for RGC [25], [26], and the sections were placed in the same medium overnight at 4°C, washed with PBS, and cover slipped.

To determine the number of RGCs, the stained flat mounts were photographed through a fluorescent microscope (BZ 8000, Keyence, Osaka, Japan). Eight areas (0.48×0.48 mm) from the 4 quadrants of the retina at a distance of 1.0 and 1.5 mm from the margin of the optic disc were photographed. All of the Tuj-1-positive cells in an area of 0.2×0.2 mm at the center of each image were counted using the NIH Image J program.

The mean density of the RGC/mm^2^ was calculated, and the loss of RGCs was determined by comparing the density of the RGCs in the retinas after optic nerve crush to that of retinas from sham operated optic nerves (n = 6 to 9). The number of RGCs was counted by one observer (MT) who was masked to whether it was from an experimental or a sham animal.

### Astrocyte Cultures from Optic Nerve

Astrocytes were isolated from the optic nerves of Wistar rats. Six rats were euthanized by CO_2_, and 6 intraorbital optic nerves were removed from the right eyes, rinsed in PBS, and incubated in 2.0 ml of EBSS containing 0.1% trypsin for 15 min. One week prior to the isolation, the right optic nerves were crushed 2 mm behind the optic discs. The nerves were further incubated in the 1.0 ml solution of 2.0% dispase from Bacillus polymyxa (Roche, Basel, Switzerland) for 15 min at 37°C. Immediately after the enzyme treatment, 10% FBS was added to the incubation medium to stop the enzyme activities. At the end of each step, the optic nerves were mechanically dissected by pipetting, and the suspension was centrifuged at 800×g for 5 min. The pellet was re-suspended in 1.0 ml of DMEM with 10% FBS and incubated with DNase for another 15 min. After dissection by pipetting and cleared by centrifugation, the pellet was re-suspended in 2.0 ml of DMEM/F-12 (Gibco, Grand Island, NY, USA) supplemented with 15% serum (each 7.5% of FBS and HS) and penicillin/streptomycin (500 units/ml) and cultured in 35 mm culture dish at 37°C in a 5.0% CO_2_/95% air atmosphere. After 24 hrs in culture, the medium was replaced by DMEM/F-12 containing the same supplements (medium A).

Subsequently, the optic nerve astrocytes were maintained in the medium A at 37°C in a 5.0% CO_2_/95% air atmosphere, and the medium was changed every 2 to 3 days. Confluence was reached after 7–10 days.

After reaching confluence, cells were released with a 0.05% trypsin solution (3.0 ml) and re-suspended in 7.0 ml of medium A and cultured in 10 cm cultured dishes. The medium was replaced by medium A on the following day and changed every 2 to 3 days. The purity of the astrocytes of cells at passage 3 was determined by immunohistochemistry and flow cytometry (FACS), and cells at passages of 3 to 6 were used in this study.

### Flow Cytometry (FACS) Analyses

In addition to the immunohistochemical studies of GFAP, the expression of GFAP by the passage 3 cells was determined by flow cytometry (Becton Dickinson, San Jose, CA, USA). Cells grown to confluence in 10 cm culture dish were harvested by trypsinization and fixed in 4.0% PFA for 1.0 hr at RT. After washing with PBS, cells were blocked in 1.0 ml of 1.0% BS in PBS-T for 1.0 hr at RT. Cells were then incubated with mouse monoclonal anti-GFAP antibody (1∶200) in PBS-T with 1.0% BS overnight at 4°C. After washing with PBS, the cells were exposed to FITC-conjugated goat anti-mouse IgG (1∶500, Merck Millipore) in 1.0 ml PBS with 1.0% goat serum for 1.0 hr at RT. After washing twice with PBS, cells (1×10^4^) were excited by a 488-nm laser light and collected in the FITC (515–545 nm) channels. The Cell Quest Acquisition and Analysis software (Becton Dickinson) was used to quantify the fluorescence signal intensities and to construct dot-density plots.

### Immunoblot

Immunoblotting was used to determine effects of simvastatin on the astroglial activation of NF-κB. TNF-α was used to induce astroglial activation of NF-κB because it is closely associated with neuro-degeneration in the optic nerve [27], [28].

Optic nerve astrocytes were grown to confluence in medium A and then cultured in serum-free DMEM for 24 hrs in the presence or absence of simvastatin (1.0 µM). This concentration was chosen because higher concentrations of simvastatin (>10 µM) were toxic to our cultured astrocytes (data not shown).

Then, the cultured astrocytes were exposed to TNF-α (50 ng/ml) in the presence or absence of simvastatin for 2 hrs. They were washed twice in PBS, harvested by scraping, and lysed with a cell lysis buffer containing phenylmethanesulfonyl fluoride (1.0 mM), pepstatin A (10 µM), leupeptin (10 µM), 0.1% SDS, 1.0% Nonidet P-40, 5.0% sodium deoxy cholate, Tris-HCl (50 mM, pH 7.6), and NaCl (150 mM). The suspension was sonicated in ice-cold water, centrifuged, and the supernatant was used to determine the total protein concentration by the Lowry method (DC Protein Assay Reagent, Bio-Rad, Hercules, CA, USA).

Samples were separated on a 10% SDS-polyacrylamide gel and transblotted onto PVDF membranes. The membranes were blocked with 5% non-fat dry milk in TBS-T (pH 7.4, 0.1% Tween 20) followed by overnight incubation at 4°C with a polyclonal p65 antibody (1∶1000, Cell Signaling) or NF-κB p65 phosphorylated at serine 536 (1∶1000; Cell Signaling). Tubulin (α-tubulin, 1∶1000; Merck Millipore) was used as an internal control. The protein bands were made visible by horseradish peroxidase-conjugated goat anti-rabbit IgG (1∶2500, Promega, Madison, WI, USA). The signals were intensified with an ECL plus Western blotting detection system (GE Healthcare, Amersham, UK). The densities of the bands of proteins were quantified with a luminescent image analyzer (LAS-3000, Fujifilm, Tokyo, Japan). The amount of phosphorylated NF-κB p65 protein expression was quantified using the equipped software (Multi Gauge version 2.02) and standardized to total NF-κB.

### Electrophoretic Mobility Shift Assay (EMSA)

The NF-κB DNA–binding activity was determined with an electrophoretic mobility shift assay (EMSA; Gel Shift Kit, Panomics, Santa Clara, CA) according to the manufacturer’s protocols. Nuclear extracts were prepared from the astrocytes with a nuclear and cytoplasmic reagent kit (Active-Motif). In brief, cultured optic nerve astrocytes were exposed to TNF-α in the presence or absence of simvastatin for 2 hrs. They were washed twice in PBS and harvested by scraping. The cells were resuspended in Hypotonic Buffer (Active-Motif), incubated for 15 min on ice, and a detergent was added. The suspension was centrifuged for 30 seconds at 14,000×g at 4°C, and the pellet was lysed in Complete Lysis Buffer (Active-Motif) and incubated for 30 min on ice. The suspension was centrifuged for 10 min at 14,000×g at 4°C, and the supernatant was used as the nuclear fraction. Then, the 2.0 µl of the nuclear extracts (2 µg/µl) was incubated with a biotin-labeled oligonucleotide containing the consensus binding sequence for NF-κB (5′-AGT TGA GGG GAC TTT CCC AGG C-3′) for 30 min at 15°C, and the transcription factor-bound oligonucleotide was separated from the unbound oligonucleotide by electrophoresis on a 6% non-denaturing polyacrylamide gel. After transfer to biodyne B nylon membrane (Thermo, Rockford, IL, USA) with a wet blot apparatus (Bio-Rad, Hercules, CA, USA), the biotin-labeled bands were made visible by horseradish peroxidase-based chemiluminescence. The densities of the bands were quantified with a luminescent image analyzer (LAS-3000). The specificity of the binding was verified by using an unlabeled consensus oligonucleotide corresponding to NF-κB binding sequence as a competitor in the binding reaction.

### Statistical Analyses

The data are expressed as means ± standard deviations (SDs). Statistical analysis was done by one-way analysis of variance (ANOVA), and if a significant change was detected, then the Scheffe or Dunnett post-hoc test was done for statistical comparisons among groups. Student’s *t* tests were used to compare 2 groups. The level of significance was set at *P*<0.05.

## Results

### Decrease in Number of RGCs after Optic Nerve Crush

**Figure 1:**
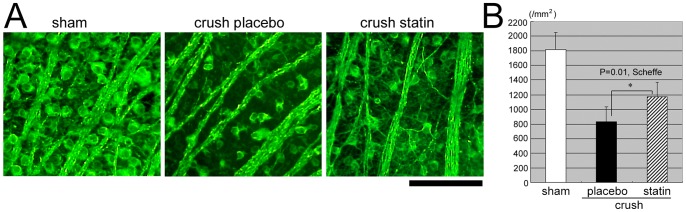
Representative photomicrographs of flat mounted retinas stained with Alexa 488-conjugated Tuj-1 antibody. A. Retinas from sham control (left), crushed optic nerves treated with placebo (middle), crushed optic nerves treated with simvastatin (right panel). Pictures were taken 1.5 mm from the optic disc margin. Bar = 100 µm. B. The density of retinal ganglion cells (RGCs/mm^2^) is quantified. Systemic simvastatin had a significant (*P* = 0.01, Scheffe) protective effect on the RGCs.

Representative photomicrographs of flat-mounted retinas taken approximately 1.5 mm from the optic disc margin are shown in Figure 1A. The RGCs are stained green with Alexa 488-conjugated Tuj-1, and the number of RGCs is fewer in animals treated by placebo (crush placebo) after crushing the optic nerve than in the sham control. The number of RGCs was significantly higher in animals that had systemic simvastatin (crush statin) compared to the vehicle-treated animals (crush placebo).

The mean ± SD number of RGCs stained by TUJ-1 antibody was 1816.3±232.6/mm^2^ in the sham operated rats (n = 6), and the mean number decreased significantly to 831.4±202.5/mm^2^ (n = 9) on day 7 in the vehicle-treated rats (Figure1B). The reduction was significantly suppressed to 1169.2±201.3/mm^2^ (n = 9) by systemic simvastatin (*P* = 0.01, Scheffe; Figure 1B).

### Immunohistochemistry

**Figure 2:**
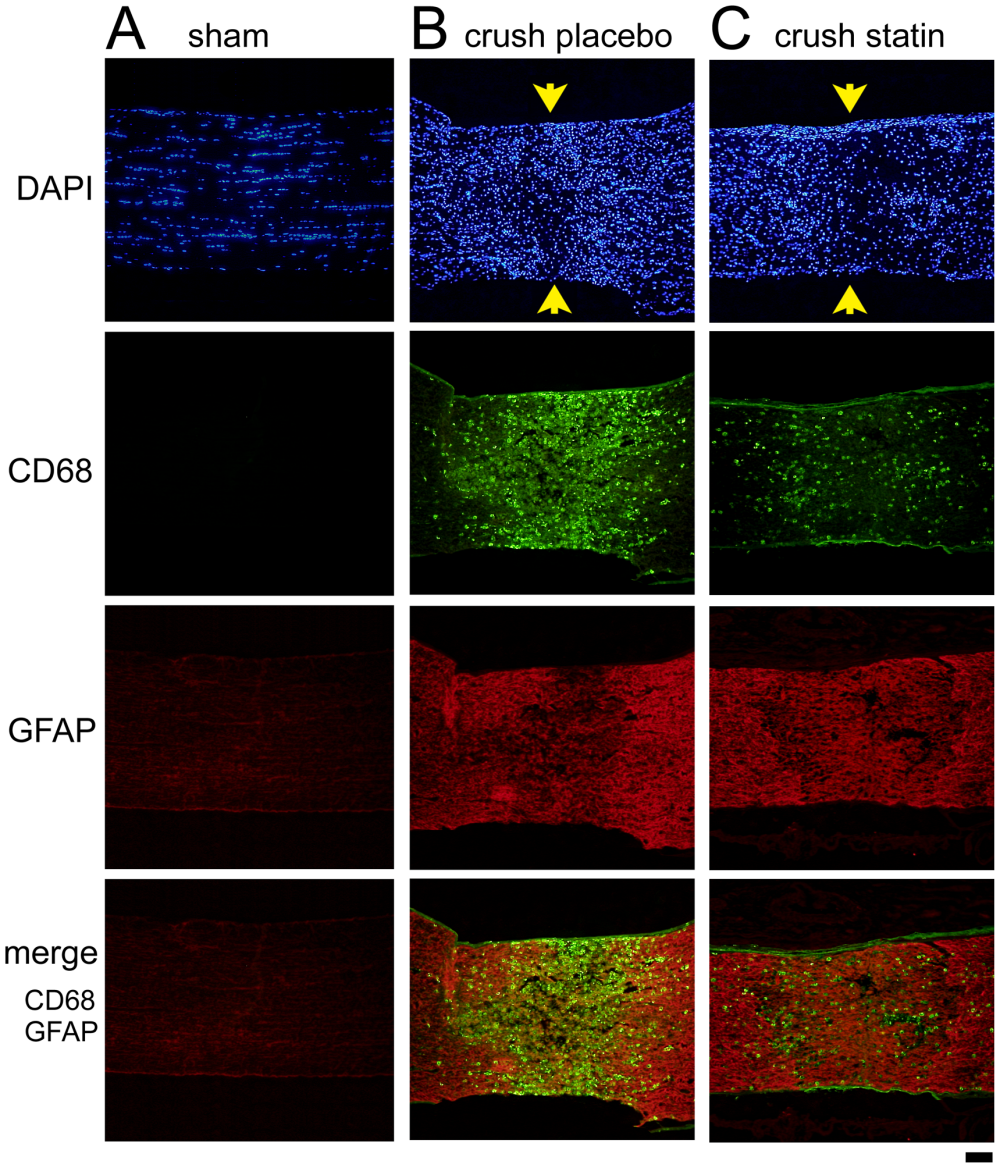
Immunohistochemistry for CD68 and GFAP expression in the crushed optic nerve on day 3. Representative photographs from 3 independent samples for each condition are presented. In each picture, 45 images were stacked using Z-scan at 0.5 µm intervals. CD68-positive cells are not visible in the sham control (A), while many CD68-positive cells (green) are present at the crushed site (arrows) in the optic nerve with placebo treatment (B). Immunoreactivity to GFAP (red) was relatively weak at the crush site, and it was intensified in the area surrounding the lesion. This relationship suggests that chemoattractant molecules are expressed in reactive astrocytes at the border of the crush site. The accumulation of CD68-positive cells and immunoreactivity to GFAP is reduced by systemic simvastatin treatment (C). CD68 staining: mouse monoclonal anti-CD68 (primary) and Alexa 488-conjugated goat anti-mouse IgG (secondary antibodies); GFAP staining: rabbit polyclonal anti-GFAP (primary) and Alexa 594-conjugated goat anti-rabbit IgG (secondary antibodies). Bar = 100 µm. A total of 9 rats were used in these analyses.

Immunohistochemistry was used to determine the inflammatory responses after the optic nerve was crushed. Representative photographs of sections immunostained for CD68 (constitutive marker expressed on microglia/macrophages) and GFAP (marker of astroglial activation) at the crushed site on day 3 are shown in Figure 2. These photographs were created using z-stacked images. Many cells that were immunopositive to CD68 (green) were detected at the crushed site where the immunoreactivity to GFAP (red) was relatively weak compared to the area surrounding the lesion (Figure 2B). Because the apparent CD68-positive cells were absent in sham control animals (Figure 2A), the accumulation of CD68 positive cells was clearly caused by crushing the optic nerve. In rats that had received systemic simvastatin, the number of CD68 positive cells appeared to be fewer (Figure 2C). The number of CD68 positive cells at crush lesions was 2054±583/mm^2^ in the vehicle treated animals (Figure 2B, crush placebo, n = 3) and 596±110/mm^2^ in the simvastatin-treated animals (Figure 2C, crush statin, n = 3). This difference was significant (*P* = 0.01, *t* test), indicating a suppression of the recruitment of microglia/macrophages.

**Figure 3:**
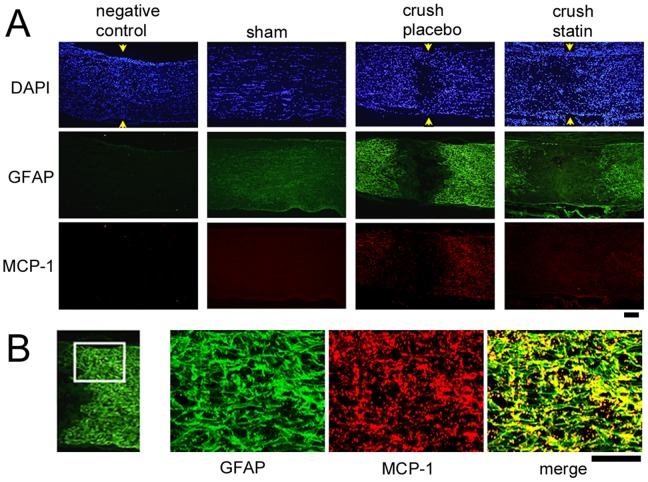
Immunohistochemistry for MCP-1 and GFAP in the optic nerve from a sham control and from experimental animals after crushing the optic nerves. Representative photographs from 3 independent samples for each condition are presented. A. Consistent with the findings shown in Figure 2, immunoreactivity to GFAP (green) was weak at the crush site, and immunoreactivity to MCP-1 (red) is also weak (crush placebo). Crushing the optic nerve intensified the immunoreactivity to MCP-1 in the area surrounding the crush site, where immunoreactivity to GFAP was increased (crush placebo) compared to the sham control. Systemic simvastatin decreases the immunoreactivities to the MCP-1 and GFAP in the area surrounding the crush site (crush statin). Images of negative control without primary antibodies were prepared from animals that underwent crushing the optic nerve. Arrows indicate crushed sites. Bar = 100 µm. B. Confocal images at the margin of the crushed site from vehicle-treated rats (square area). Immunoreactivity to MCP-1 is well co-localized with that to GFAP. MCP-1 staining: rabbit polyclonal anti-MCP-1 (primary) and Alexa 594-conjugated goat anti-rabbit IgG (secondary antibodies); GFAP staining: mouse monoclonal anti-GFAP (primary) and Alexa 488-conjugated goat anti-mouse IgG (secondary antibodies). Bar = 100 µm. A total of 12 rats were used in these analyses.

A photomicrograph showing double labeling of MCP-1 (red) and GFAP (green) at the crushed site is shown in Figure 3. Consistent with the findings shown in Figure 2, immunoreactivity to GFAP was weak at the crushed site, while it was stronger in the area surrounding the lesion (Figure 3A, crush placebo). MCP-1 is a chemokine which has been shown to attract monocytes/macrophages infiltration. Immunoreactivity to MCP-1 was also intensified at the border of the crushed site (Figure 3A, crush placebo). Merged images of GFAP and MCP-1 were taken by a confocal microscope (TCS SP8, Leica) and are shown in Figure 3B. Immunoreactivity to MCP-1 and GFAP was co-localized in these cells (Figure 3B). These findings suggest that the reactive astrocytes are the primary cells that express the MCP-1 after crushing the optic nerve. Systemic simvastatin suppressed the increase in the immunoreactivity to MCP-1 and GFAP (Figure 3A, crush statin).

**Figure 4:**
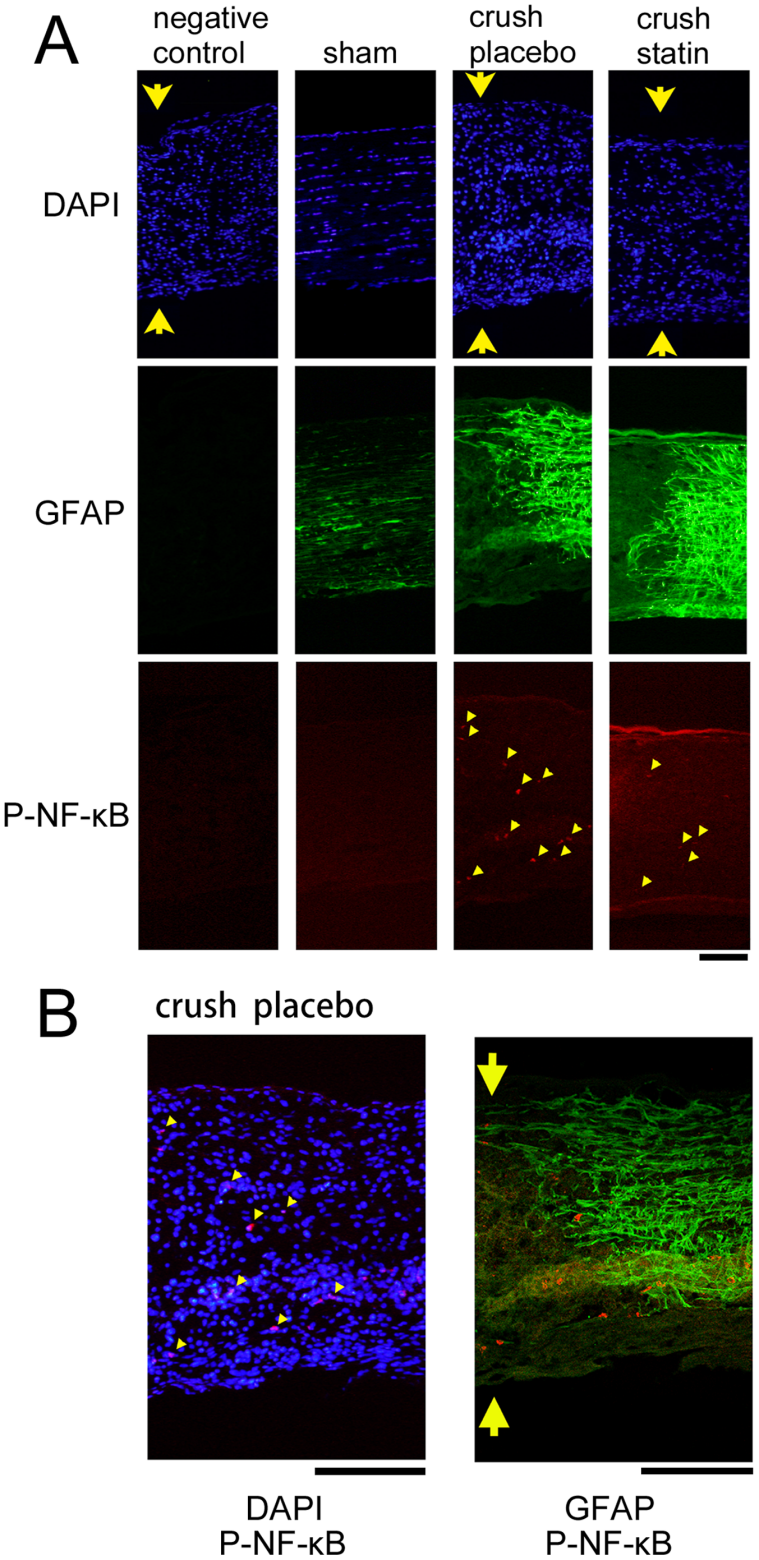
Immunohistochemistry for phosphorylated NF-κB p65 and GFAP at the margin of the crushed site of the optic nerve from sham control and from experimental animals. Representative photographs from 3 independent samples for each condition are presented. A. Phosphorylated NF-κB p65 can be detected at the border of the crushed site (arrowheads, crush placebo). Systemic simvastatin appears to decrease the number of immunopositive cells to phosphorylated NF-κB p65 (arrowheads, crush statin). There are no apparent positive cells in the sham control (sham). Images of negative control without primary antibodies were prepared from animals that underwent crushing of the optic nerve. Arrows indicate crushed site. Bar = 100 µm. B. Marged images of phosphorylated NF-κB p65, DAPI and GFAP at the margin of the crushed site (crush, placebo). Phosphorylation of NF-κB, which indicates NF-κB activation, can be detected in some nuclei (left panel). Immunoreactivity to phosphorylated NF-κB is colocalized with some GFAP positive cells at the border of the crushed site (right panel). Phosphorylated NF-κB p65 staining: rabbit polyclonal anti- phosphorylated NF-κB p65 (primary) and alexa 594-conjugated goat anti-rabbit IgG (secondary antibodies); GFAP staining: mouse monoclonal anti-GFAP (primary) and Alexa 488-conjugated goat anti-mouse IgG (secondary antibodies). Bar = 100 µm. A total of 12 rats were used in these analyses.

Because NF-κB activation is required for MCP-1 expression, immunohistochemistry on phosphorylated NF-κB p65 was also performed on the crushed optic nerves. Some immunopositive cells to phosphorylated NF-κB p65 could be detected at the edge of the crushed site (Figure 4A, crush placebo), while no positive cells were seen in the sham control animals (Figure 4A, sham). In animals treated with systemic simvastatin (Figure 4A, crush statin), the number of immunopositive phosphorylated NF-κB p65 cells appeared to be fewer than in vehicle-treated animals. The number of immunopositive cells at the border of the crushed lesions was153±33/mm^2^ in the vehicle-treated animals (Figure 4A, crush placebo, n = 3) and 71±29/mm^2^ in the simvastatin-treated animals (Figure 4A, crush statin, n = 3). This change was significant (*P* = 0.04, *t* test). A merged image showed that phosphorylated NF-κB p65 is expressed in the nuclei of some astrocytes at the border of the crushed lesion from vehicle-treated animals (Figure 4B). Immunoreactivity to TNF-α was also intensified at the border of the crushed site, which was suppressed by systemic simvastatin (Figure S1). A total of 21 rats were used in these immunohistochemical analyses.

### Alterations in Expression of Genes for ET-1, CD68, GFAP, TNF-α, and iNOS in Optic Nerve

**Figure 5:**
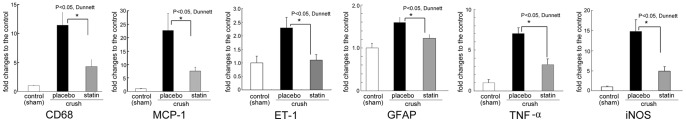
Changes in the mRNA levels of the *CD68*, *MCP-1*, *ET-1*, *GFAP*, *TNF-α*, and *iNOS* genes in the optic nerve on day 3 after crushing the optic nerves. RNA was extracted from 4(placebo). Systemic simvastatin suppresses the increase significantly (n = 6 to 8 for each condition; 22 rats were used). Data are shown as the fold changes (mean ± SD) to the sham control in the mRNA expressions.

We investigated the effects of systemic simvastatin on the neuroinflammatory events in the optic nerve. The expressions of the mRNA levels of the *CD68*, *MCP-1*, *ET-1*, *GFAP*, *TNF-α*, and *iNOS* genes in the optic nerves were examined quantitatively by RT-PCR on day 3. The mRNA levels of these genes were normalized to the 18S rRNA level (Figure 5), and the levels are presented relative to that of the sham controls (n = 6 to 8 for each condition). A total of 22 rats were used.

The mRNA level of CD68 was increased by 11.4-fold (*P = *0.001) and MCP-1 by 22.6-fold (*P* = 0.002, Scheffe) over the control level after the optic nerve was crushed in animals administered placebo. These increases were significantly depressed by systemic simvastatin administration (*P*<0.05, Dunnett), and the increase remained at 4.3-fold and 7.6-fold, respectively.

Similarly, the mRNA levels of ET-1, GFAP, TNF-α, and iNOS were increased by 2.3-fold (*P* = 0.006), 1.6-fold (*P* = 0.01), 7.0-fold (*P* = 0.01), and 14.8-fold (*P* = 0.004, Scheffe), respectively, of the sham controls on day 3. Systemic simvastatin significantly suppressed these increases (*P*<0.05, Dunnett), and the levels of these genes in animals that underwent optic nerve crush and treated with simvastatin were not different from that in sham controls (*P*>0.05, Scheffe).

There was a similar trend in the changes of mRNA levels of RhoA with other genes tested, i.e., an up-regulation after crushing the optic nerve and suppression by simvastatin (Figure S2). However, these changes were not significant in multiple comparison among the 3 groups (*P* = 0.11, ANOVA). But, when the levels were compared between experimental animals that were given simvastatin (crush statin) and vehicle (crush placebo), the mRNA levels of RhoA were significantly lower (*P* = 0.03, *t* test) in animals treated with systemic simvastatin (Figure S2).

### Astrocytes in Culture

**Figure 6:**
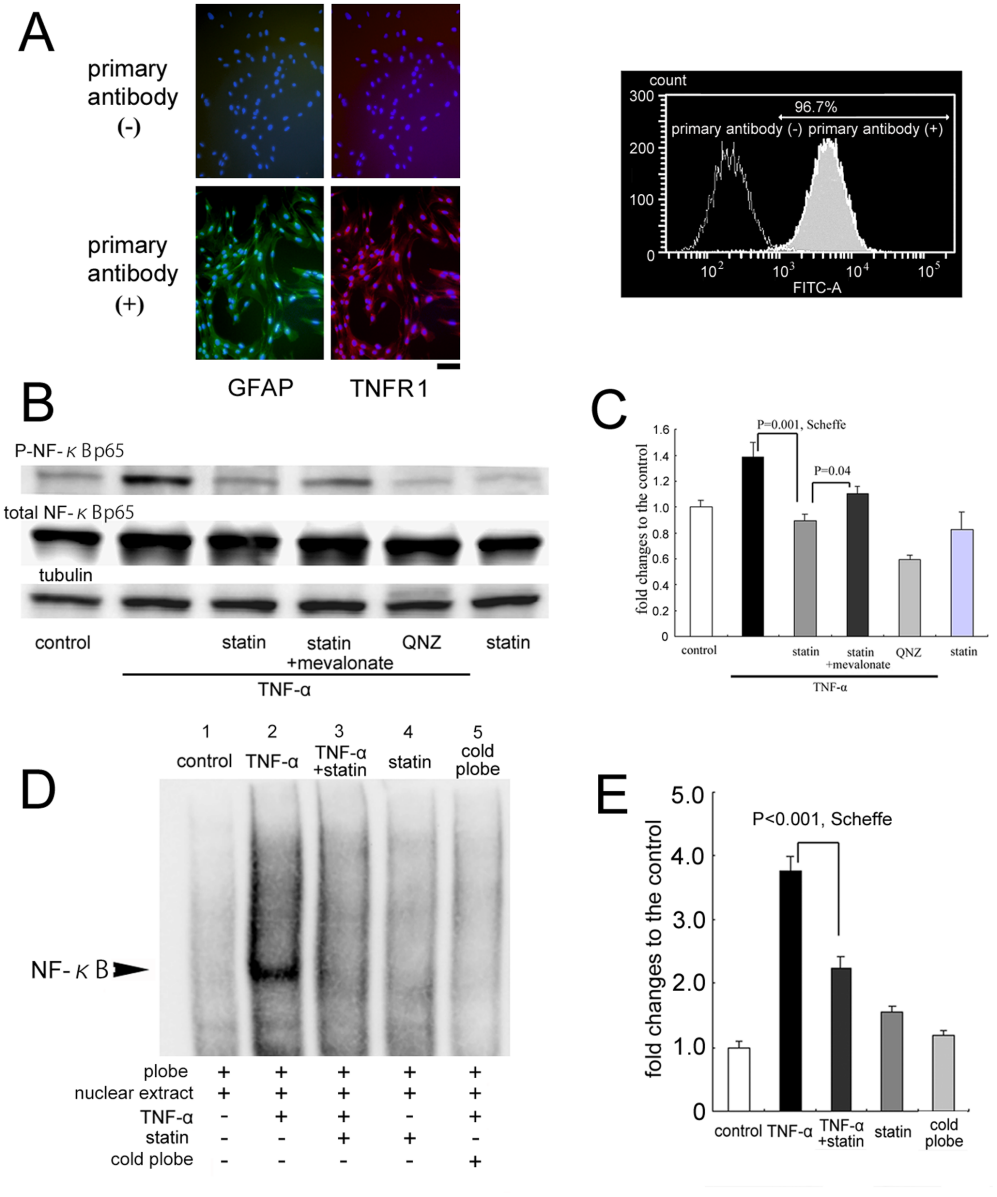
Effects of simvastatin on the TNF-α-induced NF-κB activation of optic nerve astrocytes. A. Characterization of cultured astrocytes isolated from optic nerves. Left panels; GFAP immunostaining of cultured astrocytes with DAPI nuclear counter staining. All cells are stained positively with antibody to GFAP (green). These cells are also positively stained with TNFR1 antibody (red). Bar = 100 µm. Right panel; FACS analysis of GFAP expression on our cultured astrocytes. Over 96% of the cells express GFAP. B. Representative western blot analysis of phosphorylated NF- κB p65 and total NF- κB p65. Tubulin was used as an internal control. C. Phosphorylation of NF- κB was quantified by the ratio of phosphorylated NF- κB to the total NF- κB levels. Data are shown as fold changes (mean ± SD, n = 4 for each) to the control. TNF-α induced a 1.4-fold increase of phosphorylated NF-κB from the control level, which was significantly (*P* = 0.001, Scheffe) suppressed by simvastatin. Addition of mevalonate (100 µM) significantly (*P* = 0.04, Scheffe) restored the increase. Incubation with NF- κB activation inhibitor, QNZ completely suppressed the TNF-α-induced phosphorylation of NF-κB. D. Representative EMSA analysis of NF-κB DNA binding activity. NF-κB DNA–binding activity in the nuclei of optic nerve astrocytes in the control medium (Lane 1), exposed to TNF-α (50 ng/ml) (Lane 2), exposed to TNF-α (50 ng/ml) and simvastatin (1.0 µM) (Lane 3), incubated with simvastatin (1.0 µM) alone (lane 4), NF-κB DNA–binding activity by competition EMSA with a 100-fold excess of cold NF-κB oligonucleotides (Lane 5). E. Quantification of NF-κB DNA–binding activity in the nuclei of optic nerve astrocytes cultured under different conditions. Fold changes (mean ± SD, n = 4 for each) from the control are plotted. Exposed to TNF-α (50 ng/ml) caused a 3.8 fold increase of NF-κB DNA–binding activity, which was significantly (*P*<0.001, Scheffe) suppressed by co-incubation with simvastatin (1.0 µM).

Cells isolated from intraorbital optic nerves at passage 3 were immunostained with anti-GFAP antibody and analyzed by microscopy and FACS (Figure 6A). All of the cells in the field were positively stained with anti-GFAP antibody (Figure 6A, left panels). These astrocytes were also stained with antibody to TNFR1, receptors for TNF-α with the “death-domain” motif. Additionally, FACS analysis showed that >96% of the cells were GFAP-positive indicating that our cultures were nearly a pure culture of astrocytes (Figure 6A, right panel).

### Modulation of NF-κB Activation

The effect of statin on the TNF-α-induced phosphorylation of NF-κB was determined by western blot analysis. Western blot analysis of extracts from the optic nerve astrocytes after 2 hrs of exposure to TNF-α (50 ng/ml) in the presence or absence of simvastatin is shown in Figure 6B. The level of phosphorylated NF-κB was normalized to the total NF-κB (Figure 6C). TNF-α caused a 1.4-fold increase in the phosphorylated NF-κB from the control level. Simvastatin significantly (*P* = 0.001, Scheffe) suppressed the increase, and the addition of mevalonate (100 µM) partially but significantly (*P = *0.04, Scheffe) restored the increase. The NF-κB activation inhibitor, QNZ (100 nM), decreased the level.

EMSA was also performed using nuclear extracts from the cultured optic nerve astocytes (Figure 6D). Exposure to TNF-α (50 ng/ml) induced a 3.8-fold increase of the NF-κB DNA-binding activity, while co-incubation with simvastatin (1.0 µM) significantly decreased (*P*<0.001, Scheffe) the induction by 33% (Figure 6E).

## Discussion

Our results showed that systemic simvastatin suppressed the number of CD68-positive cells at the crushed site of the optic nerve and decreased the expression of MCP-1 at the border of the lesion. Systemic simvastatin also suppressed the expression of phosphorylated NF-κB as well as the pro-inflammatory events including the up-regulation of the mRNA levels of the *CD68*, *MCP-1*, *ET-1*, *GFAP*, *iNOS*, and *TNF-α* genes. The treatment also rescued the RGCs from optic nerve injury. The results of our *in vitro* assays using cultured optic nerve astrocytes showed that simvastatin suppressed the TNF-α-induced NF-κB activation.

The up-regulation of CD68 and MCP-1 may be closely related because MCP-1 can attract microglia/macrophages to lesions in the CNS [29]. In addition, the increase in the number of macrophages was depressed in mice lacking the *MCP-1* gene after traumatic brain injury compared to wild type animals [30]. Consistent with an earlier report [31], the expressions of the *MCP-1* and *GFAP* genes were co-localized in the astrocytes in the crushed optic nerve. Thus, it is reasonable to conclude that astrocytes are the main cells that expressed MCP-1 and attracted microglia/macrophages to the crushed site.

Earlier, we demonstrated that CD68-positive cells, possibly microglia/macrophages, accumulated at the crush site and secreted ET-1, which induced reciprocal activation of astrocytes surrounding the lesion [13]. A significant reduction of other pro-inflammatory molecules including ET-1, GFAP, TNF-α, and iNOS supports the idea that simvastatin had modulated the subsequent neuroinflammatory cascades among the astrocytes and microglia/macrophages.

It has been shown that activation of glial NF-κB can regulate neuroinflammation because it drives the transcription of several pro-inflammatory molecules including TNF-α and iNOS in both microglia and astrocytes [32]. It has also been shown that suppression of astroglial NF-κB is protective to RGCs from retinal ischemia [17] and may also be effective in the treatment of optic neuritis [18]. NF-κB induces the production of chemokines including MCP-1 in astrocytes [19]. Our immunohistological study suggested that astroglial activation of NF-κB occurred at the edge of the crushed site where the MCP-1 expression was also intensified. Consistent with these findings, immunoreactivity to TNF-α was intensified at the border of the crushed site (Figure S1). Because systemic simvastatin appeared to suppress the astroglial NF-κB, it is reasonable that the suppression of the astroglial NF-κB activation could suppress neuroinflammation in the optic nerve and protect the RGCs.

We further determined quantitatively that simvastatin suppressed NF-κB activation using optic nerve astrocytes in culture. TNF-α was used to activate astroglial NF-κB because it plays a critical role in the death of RGCs after crushing the optic nerve [27]. In addition, TNF-α causes axonal degeneration in the optic nerve [28], and the expression of TNF-α increased after the optic nerves was crushed in this study. Our *in vitro* assay demonstrated that pretreatment with simvastatin suppressed the TNF-α-induced phosphorylation of astroglial NF-κB p65. It also suppressed the DNA binding activity of NF-κB. Statins inhibit HMG-CoA reductase and decrease mevalonate metabolites, which subsequently suppresses the formation of small G-proteins including those of the RAS [33] and Rho families [34]. Because the addition of mevalonate decreased the inhibitory effects of simvastatin on the TNF-α-induced activation of NF-κB, a reduction of these small G-proteins is important for the anti-inflammatory effects of simvastatin. RAS and Rho-GTPases have been reported to regulate the signal transduction of the MAP kinase family [35], which then triggers the phosphorylation and activation of NF-κB [36]. For example, the small G protein RAS p21 is important for the activation of Raf-1 [37], which in turn activates NF-κB [38]. In addition, the activity of NF-κB is stimulated by Rho independently of RAS or RAF [39]. Thus, suppression of astroglial NF-κB through a reduction of small G proteins is one possible mechanism of how systemic simvastatin protects the RGCs. Actually, the *RhoA* gene was down-regulated in the crushed optic nerve by systemic simvastatin (Figure S2). This reduction may also suggest a bioavailability of simvastatin in the optic nerve, because *RhoA* is a downstream gene of HMG-CoA reductase.

There are limitations of this study. We did not examine the effect of simvastain on retinal inflammatory responses, and simvastain may have worked in the retina and rescued the RGCs. However, we have already shown that neuroinflammation in the retina is much less than that in the crushed optic nerve [13]. Another limitation is that we did not quantify the NF-κB activities or the protein levels of the pro-inflammatory genes in the optic nerve *in vivo*. Demonstration of neuro-protection using animals with selective inhibition of astroglial NF-κB through a transgenic approach is necessary to obtain direct evidence that astroglial NF-κB plays a role in the neuro-protection. Besides, temporal changes of anti-inflammatory effects of simvastatin were not determined. Further studies are needed to clarify these issues including how simvastatin affects cytokine and chemokine networks, and the astroglial activation in the optic nerve.

In conclusion, systemic simvastatin is protective of RGCs after optic nerve injury. Our study supports the idea that simvastatin suppressed neuroinflammation in the injured optic nerve most likely by blocking the activation of astroglial NF-κB. These findings provide promising strategies against optic nerve diseases where astrogliosis prevents functional recovery.

## Supporting Information

Figure S1 **Immunohistochemistry for TNF-α at the crushed site of the optic nerve from sham control and from experimental animals.** Immunoreactivity to TNF-α was intensified, compared to the control (sham), at the border of the crushed site after crushing the optic nerve (crush placebo). Systemic simvastatin suppressed the increased immunoreactivity to TNF-α (crush statin). Images of negative control without primary antibodies were prepared from animals that underwent crushing of the optic nerve. Arrows indicate crushed site. TNF-α staining: rabbit polyclonal anti-TNF-α (primary) and alexa 594-conjugated goat anti-rabbit IgG (secondary antibodies). Bar = 100 µm.
(TIF)

Figure S2 **Changes in the mRNA levels of the RhoA gene in the optic nerve on day 3 after crushing the optic nerves.** When the levels are compared between animals that underwent optic nerve crush with systemic simvastatin (crush statin) and vehicle (crush placebo), RhoA mRNA levels are lower (P = 0.03, *t* test) in the animals treated with systemic simvastatin. Data are shown as the fold changes (mean ± SD, n = 6–8 in each condition) to the sham control in the mRNA expressions.
(TIF)

## References

[1] Eddleston M, Mucke L (1993) Molecular profile of reactive astrocytes–implications for their role in neurologic disease. Neuroscience 54: 15–36. 8515840 10.1016/0306-4522(93)90380-X PMC7130906

[2] Wilhelmsson U, Li L, Pekna M, Berthold CH, Blom S, et al (2004) Absence of glial fibrillary acidic protein and vimentin prevents hypertrophy of astrocytic processes and improves post-traumatic regeneration. J Neurosci 24: 5016–5021. 15163694 10.1523/JNEUROSCI.0820-04.2004 PMC6729371

[3] Rosenman SJ, Shrikant P, Dubb L, Benveniste EN, Ransohoff RM (1995) Cytokine-induced expression of vascular cell adhesion molecule-1 (VCAM-1) by astrocytes and astrocytoma cell lines. J Immunol 154: 1888–1899. 7530745

[4] Van Der Voorn P, Tekstra J, Beelen RH, Tensen CP, Van Der Valk P, et al (1999) Expression of MCP-1 by reactive astrocytes in demyelinating multiple sclerosis lesions. Am J Pathol 154: 45–51. 9916917 10.1016/S0002-9440(10)65249-2 PMC1853444

[5] Minagar A, Shapshak P, Fujimura R, Ownby R, Heyes M, et al (2002) The role of macrophage/microglia and astrocytes in the pathogenesis of three neurologic disorders: HIV-associated dementia, Alzheimer disease, and multiple sclerosis. J Neurol Sci 202: 13–23. 12220687 10.1016/s0022-510x(02)00207-1

[6] Nakazawa T, Takahashi H, Nishijima K, Shimura M, Fuse N, et al (2007) Pitavastatin prevents NMDA-induced retinal ganglion cell death by suppressing leukocyte recruitment. J Neurochem 100: 1018–1031. 17266736 10.1111/j.1471-4159.2006.04274.x

[7] Kawaji T, Inomata Y, Takano A, Sagara N, Inatani M, et al (2007) Pitavastatin: protection against neuronal retinal damage induced by ischemia-reperfusion injury in rats. Curr Eye Res 32: 991–997. 18027175 10.1080/02713680701649603

[8] Schmeer C, Gamez A, Tausch S, Witte OW, Isenmann S (2008) Statins modulate heat shock protein expression and enhance retinal ganglion cell survival after transient retinal ischemia/reperfusion in vivo. Invest Ophthalmol Vis Sci 49: 4971–4981. 18566458 10.1167/iovs.07-1597

[9] Krempler K, Schmeer CW, Isenmann S, Witte OW, Lowel S (2011) Simvastatin improves retinal ganglion cell survival and spatial vision after acute retinal ischemia/reperfusion in mice. Invest Ophthalmol Vis Sci 52: 2606–2618. 21245399 10.1167/iovs.10-6005

[10] Ko ML, Chen CF, Peng PH, Peng YH (2011) Simvastatin upregulates Bcl-2 expression and protects retinal neurons from early ischemia/reperfusion injury in the rat retina. Exp Eye Res 93: 580–585. 21777583 10.1016/j.exer.2011.07.003

[11] Kretz A, Schmeer C, Tausch S, Isenmann S (2006) Simvastatin promotes heat shock protein 27 expression and Akt activation in the rat retina and protects axotomized retinal ganglion cells in vivo. Neurobiol Dis 21: 421–430. 16168661 10.1016/j.nbd.2005.08.003

[12] Pahan K, Sheikh FG, Namboodiri AM, Singh I (1997) Lovastatin and phenylacetate inhibit the induction of nitric oxide synthase and cytokines in rat primary astrocytes, microglia, and macrophages. J Clin Invest 100: 2671–2679. 9389730 10.1172/JCI119812 PMC508470

[13] Tonari M, Kurimoto T, Horie T, Sugiyama T, Ikeda T, et al (2012) Blocking endothelin-B receptors rescues retinal ganglion cells from optic nerve injury through suppression of neuroinflammation. Invest Ophthalmol Vis Sci 53: 3490–3500. 22562513 10.1167/iovs.11-9415

[14] Ghosh A, Roy A, Matras J, Brahmachari S, Gendelman HE, et al (2009) Simvastatin inhibits the activation of p21ras and prevents the loss of dopaminergic neurons in a mouse model of Parkinson's disease. J Neurosci 29: 13543–13556. 19864567 10.1523/JNEUROSCI.4144-09.2009 PMC2862566

[15] Wu H, Mahmood A, Lu D, Jiang H, Xiong Y, et al (2010) Attenuation of astrogliosis and modulation of endothelial growth factor receptor in lipid rafts by simvastatin after traumatic brain injury. J Neurosurg 113: 591–597. 19895202 10.3171/2009.9.JNS09859 PMC3007601

[16] Saha RN, Pahan K (2006) Regulation of inducible nitric oxide synthase gene in glial cells. Antioxid Redox Signal 8: 929–947. 16771683 10.1089/ars.2006.8.929 PMC1963415

[17] Dvoriantchikova G, Barakat D, Brambilla R, Agudelo C, Hernandez E, et al (2009) Inactivation of astroglial NF-kappa B promotes survival of retinal neurons following ischemic injury. Eur J Neurosci 30: 175–185. 19614983 10.1111/j.1460-9568.2009.06814.x PMC2778328

[18] Brambilla R, Dvoriantchikova G, Barakat D, Ivanov D, Bethea JR, et al (2012) Transgenic inhibition of astroglial NF-kappaB protects from optic nerve damage and retinal ganglion cell loss in experimental optic neuritis. J Neuroinflammation 9: 213. 22963651 10.1186/1742-2094-9-213 PMC3490907

[19] Thompson WL, Van Eldik LJ (2009) Inflammatory cytokines stimulate the chemokines CCL2/MCP-1 and CCL7/MCP-3 through NFkB and MAPK dependent pathways in rat astrocytes. Brain Res 1287: 47–57. 19577550 10.1016/j.brainres.2009.06.081 PMC2725204

[20] Kurimoto T, Ishii M, Tagami Y, Nishimura M, Miyoshi T, et al (2006) Xylazine promotes axonal regeneration in the crushed optic nerve of adult rats. Neuroreport 17: 1525–1529. 16957602 10.1097/01.wnr.0000234749.80936.54

[21] Berkelaar M, Clarke DB, Wang YC, Bray GM, Aguayo AJ (1994) Axotomy results in delayed death and apoptosis of retinal ganglion cells in adult rats. J Neurosci 14: 4368–4374. 8027784 10.1523/JNEUROSCI.14-07-04368.1994 PMC6577016

[22] Panagis L, Thanos S, Fischer D, Dermon CR (2005) Unilateral optic nerve crush induces bilateral retinal glial cell proliferation. Eur J Neurosci 21: 2305–2309. 15869529 10.1111/j.1460-9568.2005.04046.x

[23] Indraswari F, Wang H, Lei B, James ML, Kernagis D, et al (2012) Statins improve outcome in murine models of intracranial hemorrhage and traumatic brain injury: a translational approach. J Neurotrauma 29: 1388–1400. 22233347 10.1089/neu.2011.2117

[24] Winkler BS (1972) The electroretinogram of the isolated rat retina. Vision Res 12: 1183–1198. 5043568 10.1016/0042-6989(72)90106-x

[25] Snow RL, Robson JA (1995) Migration and differentiation of neurons in the retina and optic tectum of the chick. Exp Neurol 134: 13–24. 7672033 10.1006/exnr.1995.1032

[26] Cui Q, Yip HK, Zhao RC, So KF, Harvey AR (2003) Intraocular elevation of cyclic AMP potentiates ciliary neurotrophic factor-induced regeneration of adult rat retinal ganglion cell axons. Mol Cell Neurosci 22: 49–61. 12595238 10.1016/s1044-7431(02)00037-4

[27] Tezel G, Yang X, Yang J, Wax MB (2004) Role of tumor necrosis factor receptor-1 in the death of retinal ganglion cells following optic nerve crush injury in mice. Brain Res 996: 202–212. 14697498 10.1016/j.brainres.2003.10.029

[28] Kitaoka Y, Kwong JM, Ross-Cisneros FN, Wang J, Tsai RK, et al (2006) TNF-alpha-induced optic nerve degeneration and nuclear factor-kappaB p65. Invest Ophthalmol Vis Sci 47: 1448–1457. 16565378 10.1167/iovs.05-0299

[29] Chen Y, Hallenbeck JM, Ruetzler C, Bol D, Thomas K, et al (2003) Overexpression of monocyte chemoattractant protein 1 in the brain exacerbates ischemic brain injury and is associated with recruitment of inflammatory cells. J Cereb Blood Flow Metab 23: 748–755. 12796723 10.1097/01.WCB.0000071885.63724.20

[30] Semple BD, Bye N, Rancan M, Ziebell JM, Morganti-Kossmann MC (2010) Role of CCL2 (MCP-1) in traumatic brain injury (TBI): evidence from severe TBI patients and CCL2−/− mice. J Cereb Blood Flow Metab 30: 769–782. 20029451 10.1038/jcbfm.2009.262 PMC2949175

[31] Glabinski AR, Balasingam V, Tani M, Kunkel SL, Strieter RM, et al (1996) Chemokine monocyte chemoattractant protein-1 is expressed by astrocytes after mechanical injury to the brain. J Immunol 156: 4363–4368. 8666808

[32] Hayden MS, Ghosh S (2008) Shared principles in NF-kappaB signaling. Cell 132: 344–362. 18267068 10.1016/j.cell.2008.01.020

[33] Casey PJ, Solski PA, Der CJ, Buss JE (1989) p21ras is modified by a farnesyl isoprenoid. Proc Natl Acad Sci U. S. A. 86: 8323–8327. 10.1073/pnas.86.21.8323 PMC298273 2682646

[34] Rattan R, Giri S, Singh AK, Singh I (2003) Rho A negatively regulates cytokine-mediated inducible nitric oxide synthase expression in brain-derived transformed cell lines: negative regulation of IKKalpha. Free Radic Biol Med 35: 1037–1050. 14572607 10.1016/s0891-5849(03)00459-3

[35] Pazman C, Mayes CA, Fanto M, Haynes SR, Mlodzik M (2000) Rasputin, the Drosophila homologue of the RasGAP SH3 binding protein, functions in ras- and Rho-mediated signaling. Development 127: 1715–1725. 10725247 10.1242/dev.127.8.1715

[36] Bian ZM, Elner SG, Yoshida A, Kunkel SL, Su J, et al (2001) Activation of p38, ERK1/2 and NIK pathways is required for IL-1beta and TNF-alpha-induced chemokine expression in human retinal pigment epithelial cells. Exp Eye Res 73: 111–121. 11428868 10.1006/exer.2001.1019

[37] Kikuchi A, Williams LT (1994) The post-translational modification of ras p21 is important for Raf-1 activation. J Biol Chem 269: 20054–20059. 8051091

[38] Li S, Sedivy JM (1993) Raf-1 protein kinase activates the NF-kappa B transcription factor by dissociating the cytoplasmic NF-kappa B-I kappa B complex. Proc Natl Acad Sci U. S. A (90) 9247–9251. 10.1073/pnas.90.20.9247 PMC47544 8415686

[39] Perona R, Montaner S, Saniger L, Sanchez-Perez I, Bravo R, et al (1997) Activation of the nuclear factor-kappaB by Rho, CDC42, and Rac-1 proteins. Genes Dev 11: 463–475. 9042860 10.1101/gad.11.4.463

