# Supplementary figures and images for: Systemic Simvastatin Rescues Retinal Ganglion Cells from Optic Nerve Injury Possibly through Suppression of Astroglial NF-κB Activation
Source: PLoS One. 2014 Jan 2;9(1):e84387. doi: 10.1371/journal.pone.0084387 (PMC3879303; doi:10.1371/journal.pone.0084387)

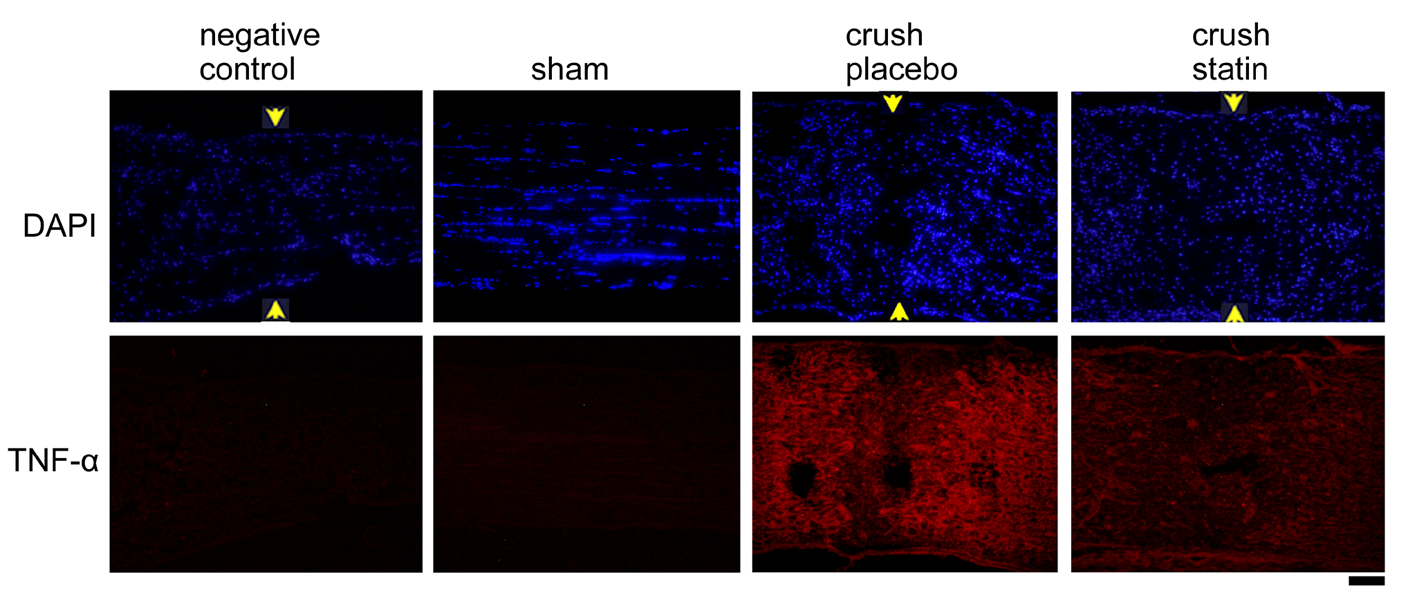

Supplement: Figure S1 — Immunohistochemistry for TNF-α at the crushed site of the optic nerve from sham control and from experimental animals. Immunoreactivity to TNF-α was intensified, compared to the control (sham), at the border of the crushed site after crushing the optic nerve (crush placebo). Systemic simvastatin suppressed the increased immunoreactivity to TNF-α (crush statin). Images of negative control without primary antibodies were prepared from animals that underwent crushing of the optic nerve. Arrows indicate crushed site. TNF-α staining: rabbit polyclonal anti-TNF-α (primary) and alexa 594-conjugated goat anti-rabbit IgG (secondary antibodies). Bar = 100 µm. (TIF) [file pone.0084387.s001.tif]

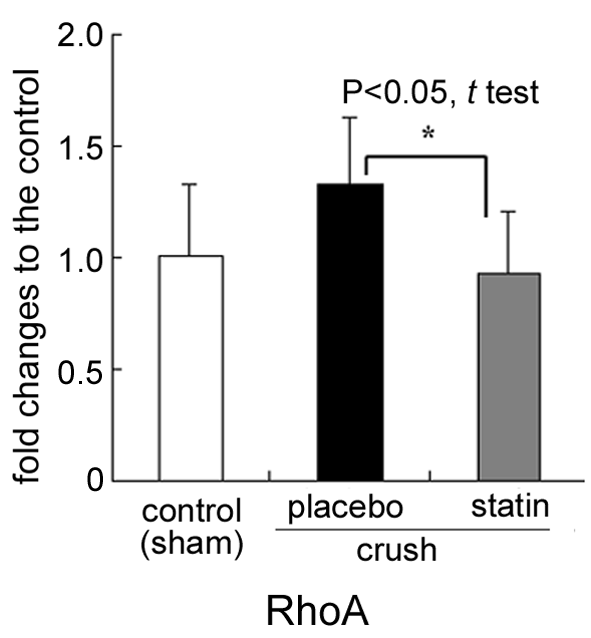

Supplement: Figure S2 — Changes in the mRNA levels of the RhoA gene in the optic nerve on day 3 after crushing the optic nerves. When the levels are compared between animals that underwent optic nerve crush with systemic simvastatin (crush statin) and vehicle (crush placebo), RhoA mRNA levels are lower (P = 0.03, t test) in the animals treated with systemic simvastatin. Data are shown as the fold changes (mean ± SD, n = 6–8 in each condition) to the sham control in the mRNA expressions. (TIF) [file pone.0084387.s002.tif]
